# Effect of calcitonin gene-related peptide antagonist on the cardiovascular events, mortality, and prostaglandin E_2_ production by nitrate-induced tolerant rats with acute myocardial infarction

**DOI:** 10.1186/s13167-016-0055-5

**Published:** 2016-03-08

**Authors:** Tamar Kezeli, Tamari Rukhadze, Nikoloz Gongadze, Galina Sukoyan, Nino Dolidze, Mariam Chipashvili, Makrine Mirziashvili

**Affiliations:** 1 Department of Pharmacology, Faculty of Medicine, I. Javakhishvili Tbilisi State University, 2 Chiaureli str., 0159 Tbilisi, Georgia; 2 Department of Medical Pharmacology and Pharmacotherapeutics, Tbilisi State Medical University, 33 Vazha-pshavela ave., 0177 Tbilisi, Georgia; 3 International Scientific Centre of Introduction of New Biomedical Technology, Assignee of the NV Karsanov Research Centre of Medical Biophysics and Introduction of New Biomedical Technology, Kayrskaya str.19, 0137 Tbilisi, Georgia; 4 Grigol Robakidze University, 6 Jano Bagrationi str., 0160 Tbilisi, Georgia

**Keywords:** Predictive preventive personalized medicine, Acute myocardial infarction, Nitroglycerine tolerance, Calcitonin gene-related peptide antagonist, Ventricular arrhythmias, Baroreflex sensitivity, Mortality, Hemodynamic, Prostaglandin E_2_

## Abstract

**Background:**

Anti-ischemic effects of NO releasing by nitroglycerin (NTG) and the release of calcitonin gene-related peptide (CGRP) are involved in the decrease of vascular remodeling in different cardiovascular diseases. Using a nitrate-free period is still generally required to prevent nitrate tolerance and should be used as the first-line option to maintain adequate symptom control and on an individual basis. Personalized anti-ischemic concerns require the urgent change of paradigm from interventional measures to predictive, preventive, and personalized treatment with organic nitrates and its combination with drugs that may improve prognosis and drugs that can be added for patients who remain symptomatic despite therapy with the other classes of agents. The purpose of this study was to evaluate the influence of human calcitonin gene-related peptide antagonist (CGRP_8-37_) on cardiohemodynamic events, prostaglandin E_2_ (PGE_2_) plasma concentration, the severity of ventricular arrhythmias, and mortality occurring during acute myocardial infarction (AMI) in NTG-tolerant and nontolerant rats.

**Methods:**

In the pilot study of efficacy of calcitonin gene-related peptide antagonist (CGRP_8-37_), 58 male Wistar rats were included. All procedures were performed according to protocols approved by the General Animal Care and Use Committee. Adult male rats underwent surgery to induce AMI by ligating the left anterior descending coronary artery or SHAM. ECG was used to confirm myocardial ischemia. In each experiment, a rat was maintained under anesthesia for the duration of the experiment. At the end of the experiment, the rat was killed by an overdose of pentobarbital. All animals in accordance with the received pharmacological agent were randomized into three groups: I—received only NTG, 50 mg/kg daily, s.c. injections b.i.d. 3 days prior to AMI; II—received NTG by the same dose, route, and frequency of administration + CGRP antagonist (CGRP_8-37_), 10 μg/kg two times daily by a similar period of administration; and III—served as control (C) group without preliminary tolerance to NTG.

**Results:**

Subcutaneous injections of NTG (50 mg/kg) 30 min prior to AMI in NTG-tolerant animals (group I) and in NTG-tolerant rats + CGRP antagonist (group II) caused minor changes in blood pressure and heart period that was accompanied before NTG s.c. administration with blunted baroreflex sensitivity in response to i.v. administration of sodium nitroprusside in these groups of rats (0.66 ± 0.05 and 0.56 ± 0.04 ms/mmHg, *P* < 0.05, respectively) in comparison to C (group III) animals (0.9 ± 0.1 ms/mmHg). AMI 1 h duration was associated with a high incidence of ventricular arrhythmia and significant mortality in group I (70 %) and especially in group II (90 %) animals at 72 h after reperfusion as compared with group III rats (56 %), that correlated to a decrease of PGE_2_ plasma content in group II (2.2 ± 0.4 ng/ml, *P* < 0.001) and group I (3.6 ± 0.2 ng/ml, *P* < 0.01) vs. control group of rats (4.8 ± 0.3 ng/ml).

**Conclusions:**

CGRP could be involved in the mechanism of nitrate tolerance via the inhibition of release of the potent vasodilator CGRP leading to exacerbation of acute myocardial ischemia. The influence of CGRP antagonist could enhance this condition.

## Background

Coronary artery disease (CAD) is the predominant cause of death in most developed countries. The clinical challenge in the treatment of angina and acute coronary syndrome and possibly less of an issue of chronic ischemic heart disease is the development of tolerance to nitrate therapy. No one therapy that has been studied seems convincing as providing a solution to nitrate tolerance either because conflicting results have been published or because data are too limited to make specific recommendations. Recent pre-clinical and clinical studies have established that, rather than a homogeneous class, organic nitrates represent a heterogeneous group of substances that differ greatly with respect to side effects such as the induction of nitrate tolerance and endothelial dysfunction. Furthermore, studies of alternative options to avoid or manage nitrate tolerance have used hemodynamic endpoints and not clinical endpoints, so their results are difficult to translate into clinical practice. Using a nitrate-free period is still generally required to prevent nitrate tolerance and should be used as the first-line option to maintain adequate symptom control and on an individual basis [1]. Thus, personalized anti-ischemic concerns require the urgent change of paradigm from interventional measures to predictive, preventive, and personalized treatment with organic nitrates and its combination with drugs that may improve prognosis (such as statins, ACE inhibitors, and aspirin, or, in the secondary prevention, beta-receptor blockers) and drugs that can be added for patients who remain symptomatic despite therapy with the other classes of agents (such as calcium antagonists, beta-receptor blockers). Some experimental and clinical studies have suggested that long-term nitrate treatment does not improve or may even worsen cardiovascular mortality, possibly due to the development of vascular nitrate tolerance.

The rapid development of tolerance against the hemodynamic effects of nitrates during sustained therapy limits their clinical application [2–4]. Nitroglycerin, which helps impaired cardiac function as it is converted to nitric oxide, is used worldwide to treat patients with various ischemic and congestive cardiac diseases, including angina pectoris. The mechanism underlying nitroglycerin-induced vascular smooth muscle relaxation involves NO production, which stimulates both soluble guanylate cyclase (sGC) and release of prostacyclin or prostaglandin E (PGE). Activation of sGC and adenylyl cyclase (AC) increases the formation of the second messengers cyclic guanosine monophosphate (cGMP) and cAMP. Subsequent activation of cGMP- and cAMP-dependent kinase (cGK and cAK) leads to vasorelaxation by phosphorylation of vasodilator-stimulated phosphoprotein (p-VASp) at serine 239 [5–9]. Increased generation of oxygen free radicals during nitrate therapy may be another important mechanism of tolerance [7, 10, 11]. Long-term treatment with NTG stimulates the production of reactive oxygen species such as peroxynitrite (ONOO^−^), which may in turn induce tolerance and endothelial dysfunction [7, 12–14]. Previous studies have suggested that NTG can evoke the release of calcitonin gene-related peptide (CGRP), which is widely distributed in vascular tissues (and is a potent vasodilator), from capsaicin-sensitive sensory nerves [3]. It was shown that CGRP mediates the depressor effect and vasodilatation produced by NTG via the cGMP pathways [15, 16]. Recent evidence suggest that diminished availability of CGRP is associated with nitrate tolerance [10, 16]. The mechanisms underlying NTG-induced vascular smooth muscle relaxation encompassing sGC pathway and release of vasodilating prostaglandins. At the same time, the CGRP, which is a potent vasodilator and mediates the depressor effect and vasodilation, is produced by NTG via the cGMP pathways. Based on these biochemical pathways, the CGRP antagonist (human calcitonin gene-related peptide antagonist (CGRP_8-37_)) may contribute to NTG-induced tolerance, cardiohemodynamic events, and mortality during myocardial infarction.

Anti-ischemic effects of NO releasing by NTG and the release of CGRP are involved in the decrease of vascular remodeling in different cardiovascular diseases. The purpose of this study was to evaluate the influence of the CGRP (human CGRP_8-37_, Sigma-Aldrich) antagonist on cardiohemodynamic events, prostaglandin E_2_ (PGE_2_) plasma concentration, the severity of ventricular arrhythmias (VA), and mortality occurring during acute myocardial infarction (AMI) in nitroglycerin-tolerant and nitroglycerin-nontolerant rats.

## Methods

In the pilot study of the efficacy of calcitonin gene-related peptide antagonist (CGRP_8-37_), 58 male Wistar rats weighing 250–300 g were included. All animals were handled in compliance with the European Convention on Animal Care and received humane care in accordance with the Principles of Laboratory Animal Care and the Guide for the Care and Use of Laboratory Animals (published in the Official Daily N. L358/1-358/6, 18 December 1986), and the US National Institutes of Health (Guide for the Care and Use of Laboratory Animals, NIH publication no. 85-23) and all procedures were performed according to protocols approved by the General Animal Care and Use Committee of the Department of Pharmacology of I. Javakhishvili Tbilisi State University, Department of Medical Pharmacology and Pharmacotherapeutics of Tbilisi State Medical University, and International Scientific Centre of Introduction of New Biomedical Technology, Tbilisi, Georgia. Animals which were maintained and fed ad libitum on the standard normal-protein diet and had free access to water were used in the study. They were housed individually in a humidity- and temperature-controlled room with a 12-h light/dark cycle at a temperature of 22 °C. Adult male rats underwent surgery to induce acute myocardial infarction (AMI) by 40 % ligation of the left anterior descending coronary artery or SHAM, and a 72-h recovery period was given in order to fully evaluate the mortality rate. ECG tracing in lead II was obtained permanently before induction of AMI and up to 60 min postinfarction to confirm myocardial ischemia. In each experiment, a rat was maintained under anesthesia during experiments. At the end of the experiment, the rat was killed by an overdose of sodium pentobarbital. Tolerance was induced by treatment with NTG (50 mg/kg daily, s.c. injections b.i.d.) for 3 days and was confirmed in previous experiments by a reduction in hypotensive responses and reflex tachycardia to intravenous NTG, that have been measured by tail-cuff method and cardiotachometer, respectively. All animals in accordance with the received pharmacological agent were randomized into three groups: I—received only nitroglycerin (NTG, 50 mg/kg daily, s.c. injections b.i.d.) 3 days prior to AMI; II—received NTG by the same dose, route, and frequency of administration + CGRP antagonist (CGRP_8-37_, Sigma-Aldrich), 10 μg/kg two times daily by a similar period of administration; and III—served as control (C) group without preliminary tolerance to NTG. After 3 days, the rats were anesthetized with sodium pentobarbital (60 mg/kg). A polyethylene catheter was implanted into the right femoral artery and connected to a blood pressure transducer for measuring blood pressure (BP) with an electromanometer and heart period (HP) with a cardiotachometer. Baroreflex sensitivity (BRS) was assessed by measuring HP in response to reduction in BP induced by i.v. sodium nitroprusside 10 μg/kg. There was a highly significant linear relationship between HP and BP for each experimental treatment (*r* values ranged from 0.84 to 0.98). The slope of relationship between BP and HP was used as an index of BRS [11]. NTG (50 mg/kg) s.c. was administered 30 min prior to coronary artery ligation. At 60 min postinfarction for determination of PGE_2_ plasma content, 1 ml of blood was drawn from the femoral artery and was aliquoted in plastic tubes (100 μl/tube) and then treated with the Ca^2+^ ionofore A 23187 (50 μl M) [17]. Fifteen minutes later, the tubes were centrifuged (1500*g*, 4 °C, 5 min) and the plasma obtained was stored (−40 °C) until measurement of PGE_2_ were performed using enzyme immunoassay kits (EIA) (R&D System, Minneapolis, MN).

Results are reported as mean ± standard deviation, and Student’s *t* test was used to compare mean values. Statistical differences were considered significant when *P* < 0.05. Means of the hemodynamic and baroreflex variables were compared between groups and throughout protocol by two-way analysis of variance (ANOVA) with repeated measures.

## Results

### Mortality after acute myocardial infarction in different groups of animals

The analysis of lethal outcome during MI showed the highest incidence of mortality in group I and especially group II of rats (Table 1). In the case of NTG-tolerant rats, the lethal outcome reached 70 %, while in group II of the animals, the mortality was 90 %. Most animals from the abovementioned groups died during 0–24 h in comparison to nontolerant rats in which NTG s.c. injection partially prevents the development of severe ventricular arrhythmias and decreased incidence of mortality (56 %) vs. 70 and 90 % in groups I and II of the animals, respectively, during 0–24 h. Eventually, according to our results, it may be suggested that in NTG-tolerant rats, the vasorelaxation properties of s.c. NTG is markedly reduced, which is associated with the reduction of cardiac rhythm compensatory enhancement and diminution in BRS.

**Table 1:** The influence of NTG on the mortality in NTG-tolerant rats with acute myocardial infarction

Group of animals	Mortality		
	0–24 h	24–48 h	48–72 h
Control (*n* = 18)	5	2	3
	Total = 10/18 (56 %)		
I—NTG-tolerant (*n* = 20)	11	3	–
	Total = 14/20 (70 %)		
II—NTG-tolerant + CGRP antagonist (*n* = 20)	18	–	–
	Total = 18/20 (90 %)		

Note: comparison the same as Table 3

### Hemodynamics response and baroreflex sensitivity in NTG-tolerant rats with acute myocardial infarction

A nitrate tolerance state means that after a prolonged, continuous, or high-dose nitrate treatment, the clinical or hemodynamic response to organic nitrates (i.e., vasodilation with subsequent decrease in BP or relief of angina pain) is attenuated or abolished. As shown in Table 2 in our experiments, s.c. injection of NTG in a dose of 50 mg/kg 30 min prior to AMI caused a distinctive alteration in hemodynamic parameters of different groups of rats. The hypotensive reaction was markedly expressed in nontolerant, control group of animals with a corresponding decrease in arterial pressure in average—28.4 ± 2.5 mmHg (*P* < 0.05). BP reduction in these animals was accompanied with reflex tachycardia and diminution in heart period (HP)—25.6 ± 3.8 ms (*P* < 0.05) below the control level. Such hemodynamic changes in nontolerant rats were correlated with markedly expressed BRS (0.9 ± 0.1 ms/mmHg) with reduction in HP in response to decreases in BP obtained by sodium nitroprusside administration before NTG s.c. injection. In contrast to nontolerant rats, s.c. injection of NTG in NTG-tolerant animals (group I) and in NTG-tolerant rats + CGRP antagonist caused less decrease in BP and HP which was combined with blunted BRS (0.66 ± 0.05 and 0.58 ± 0.04 ms/mmHg, *P* < 0.05 in groups I and II of rats, respectively) in response to reduction in BP.

**Table 2:** The NTG-induced cardiohemodynamic alterations in different groups of rats with acute myocardial infarction

Parameters	Animal groups					
	Control (nontolerant), *n* = 18		I—NTG-tolerant, *n* = 20		II—NTG-tolerant + CGRP antagonist, *n* = 20	
	a	b	a	b	a	b
BP (mmHg)	115 ± 6	−28.4 ± 2.5	109 ± 5	−16.2 ± 1.6*	110 ± 8	−8.8 ± 1.2***^###^
HP (ms)	158 ± 7	−25.6 ± 3.8	151 ± 5	−11.0 ± 1.2*	147 ± 6	5.2 ± 1.4***^###^
BRS (ms/mmHg^)^	0.9 ± 0.1		0.66 ± 0.05**		0.58 ± 0.04**	

*BP* blood pressure, *HP* heart period, *BRS* baroreflex sensitivity, *a* initial values, *b* after NTG injection, *n* number of animals

Difference with the control group: *p < 0.05; **p < 0.01; ***p < 0.001; difference with the I—NTG-tolerant group: ^###^ *p* < 0.001

### Changes in ECG parameters in NTG-tolerant rats with acute myocardial infarction

The estimation of ECG showed that AMI was accompanied by a high incidence of ventricular arrhythmias (VA) in a form of ventricular tachycardia (VT) and ventricular premature beats (VPBs). Myocardial ischemia induced the elevation of ST segment with its following inversion, which in majority of groups I and II of rats unlike control group of animals (Table 3) was associated with VT in which the duration in NTG-tolerant (19 ± 4 s (*P* < 0.001) and especially NTG-tolerant + CGRP antagonist rats 24 ± 6 s (*P* < 0.001) significantly exceeds such episodes in nontolerant (control group) rats (9 ± 2 s). The average number of evoking VPBs in each animal of groups I and II significantly exceed those in the control group of rats (520 ± 38 and 668 ± 52 vs. 440 ± 30, respectively). The severe arrhythmias in groups I and II of rats sometimes transformed into ventricular fibrillation with lethal outcome, while in nontolerant rats, its episodes were absent.

**Table 3:** The influence of NTG on arrhythmias in NTG-tolerant rats with acute myocardial infarction

Group of animals	Monitoring started after 1 h of AMI and lasted during 1 h					
	VT		VPBs		VF	
	Number of animals having VT	Average duration in seconds in each animal	Number of animals having VPBs	Average number of VPBs in each animal	Number of animals having VF	Average duration in seconds in each animal
Control, *n* = 18	6	9 ± 2	12	440 ± 30	–	–
I—NTG, *n* = 20	11	19 ± 4**	7	520 ± 24*	2	66 ± 12
II—NTG + CGRP antagonist, *n* = 20	13	24 ± 6**	4	668 ± 52**^#^	3	85 ± 14^#^

*VT* ventricular tachycardia, *VPBs* ventricular premature beats, *VF* ventricular fibrillation, *n* number of animals in each group

Difference with the control group: *p < 0.05; **p < 0.01; difference with the I—NTG-tolerant group: ^#^ *p* < 0.05

### PGE_2_ generation in NTG-tolerant rats with acute myocardial infarction

**Fig. 1:**
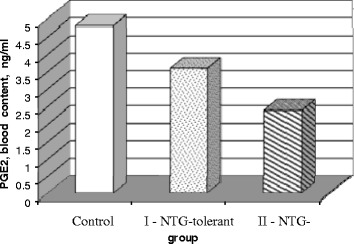
Effect of NTG (50 mg/kg) s.c. 30 min prior to AMI on the blood content of PGE_2_ in different groups of rats with myocardial ischemia. *C* control group (*n* = 18), *I* NTG-tolerant (*n* = 20), *II* NTG-tolerant + CGRP antagonist (*n* = 20)

Because one of the markers of adaptive (positive) reserve of ischemic/hypoxic damage of myocardium is the upregulation of COX-2 with a subsequent increment of PGE_2_ generation, we have determined the content of PGE_2_ as a cytoprotective heart prostanoid in hypoxic conditions in NTG-tolerant animals after AMI [2]. The content of PGE_2_ in blood samples obtained at 60 min of AMI revealed a great difference in experimental animals (Fig. 1). Its maximal concentration has been identified in nontolerant rats (group C) with mean values 4.8 ± 0.3 ng/ml vs. NTG-tolerant (3.6 ± 0.2 ng/ml) and NTG-tolerant + CGRP antagonist (2.2 ± 0.4 ng/ml) groups of animals, respectively (*P* < 0.05), that proves the reduction of PGE_2_ release in NTG-tolerant and predominantly in NTG-tolerant + CGRP antagonist group of animals. PGE_2_ could act as a beneficial modulator in the myocardium and prevents a major injury of it.

Diminished release of PGE_2_ and CGRP may be partially accountable for such alteration because in NTG-tolerant rats and NTG-tolerant + CGRP antagonist animals, PGE_2_ blood content is profoundly reduced in comparison to nontolerant rats. Along with such changes, the cardioprotective properties of s.c. NTG in the case of tolerant condition is significantly reduced, that was proved by more incidence of severe ventricular arrhythmias and high frequency of mortality in these groups of animals to compare with nontolerant rats.

## Discussion

Tolerance is an important consideration in the use of nitrates [18, 19], and NTG therapy has been shown to be associated with activation of neurohormonal vasoconstrictor forces. NTG-induced decreases in BP caused baroreflex stimulation leading to a variety of neurohumoral adjustments, which include increases in catecholamine levels and release rates and increases in plasma vasopressin [16], plasma renin activity, and angiotensin II and aldosterone levels [12, 20]. The intrinsic mechanism of angiotensin II (AngII) and sympathetic nerve reflexes, baroreflex sensitivity, and sensory nerve activator homeostasis maintenance includes the sensory nerve-derived CGRP release stimulation. CGRP does not play a primary role in the regulation of basal blood pressure (BP) in normal individuals but is suggested to have protective properties, in cardiovascular disease, including attenuation of vascular smooth muscle proliferation, hyperplasia, and stimulation of endothelial cell proliferation and endothelial progenitor cells [21]. However, there is little evidence of detailed analysis involving the ongoing influence of endogenous CGRP on vascular remodeling, especially with regard to NO and oxidative stress pathways. In our experiments in NTG-tolerant animals and especially in NTG-tolerant rats + CGRP antagonist, subsequent NTG injections 30 min prior to AMI did not cause significant changes in BP and HP that was accompanied with a reduction of baroreflex sensitivity in comparison to control group of rats. These results correspond to the findings of the abovementioned authors, and other investigators demonstrated that long-term continuous transdermal NTG therapy has been associated with altered autonomic neural function, including impaired baroreflex activity and increased sensitivity to receptor-dependent vasoconstrictors such as serotonin, phenylephrine, angiotensin II, and thromboxan [16, 20]. A marked increase in intravascular volume, secondary to the transvascular shift of fluid and/or to aldosterone-mediated salt and water retention, has also been observed in patients treated with NTG. Although these changes could attenuate the preload effect of NTG, evidence suggest that these mechanisms are not sufficient to fully explain the loss of nitrate effectiveness, and the prognostic implications of these changes, other mechanisms of tolerance, and hypothesis that explained all these alterations had to be sought [10, 16]. Evidence of NTG-induced increased reactive oxygen species (ROS) production in humans was obtained ex vivo in arterial segments and in blood or platelets taken from patients rendered tolerant to NTG [7, 13, 18, 22, 23]. NTG tolerance was also associated with increased markers of free radical-induced lipid peroxidation such as cytotoxic aldehydes and isoprostanes [23, 24] and esterified 8-epi-PGE_2α_ [24] and with a mild reduction in the responsiveness to the NO donor sodium nitroprusside in healthy volunteers [15], which might also be compatible with ROS-mediated interference with NO signaling [16]. From these findings, the authors proposed that the existence of a unifying hypothesis that founded on the concept of NTG-induced increased oxidative stress could be compatible with the multiple different observations associated with long-term nitrate treatment [19, 20]. Existing observations include the demonstration that the vasodilator actions of NTG are, at least in part, endothelium-dependent and that in the presence of increased oxidative stress, NTG can actively cause constriction of resistance vessels via release of PGF_2α_ and TXA_2_ [13, 24, 25]. In our experiments in groups I and II of the animals, a marked decrease of PGE_2_ content in blood samples obtained at 60 min of AMI has been established in comparison to group III of rats that proves the reduction of PGE_2_ release in NTG-tolerant and predominantly in the NTG-tolerant + CGRP antagonist group of animals. These findings indicated the reduction releases of vasodilating PGE_2_ in NTG-tolerant rats. At the same time, there is controversial data concerning the anti-ischemic effects of NTG in NTG-tolerant rats [13, 25]. The preferential effect of nitrates is the dilation of the systemic veins; however, they also cause arterial vasodilation leading to decreased afterload [26–28]. In addition, a direct cardioprotective effect of nitrate has been implicated [3, 5]. It has been shown that a non-vasodilator concentration of NTG exerts a direct myocardial anti-ischemic effect independent of its vascular actions in isolated rat hearts and in conscious rabbits which is not diminished by the development of vascular tolerance to NTG [4, 29]. However, the involvement of cGMP in these cardiac effects of nitrates has been questioned [3]. These studies suggest that the in vivo cellular mechanisms of action of organic nitrates are probably more complex than it was previously believed, and it is possible that nitrates trigger different biochemical mechanisms in the heart than in the systemic vascular elements [7, 8, 30]. As an alternative, the mechanism triggered by nitrate treatment may be the same in the different tissues; however, these pathways are regulated in a distinct way [26]. Several studies have demonstrated that despite the loss of preconditioning induced by rapid ventricular pacing in rabbits that made them tolerant to the vasodilator effect of NTG, a direct anti-ischemic effect of NTG was preserved [7, 26, 31–33]. Moreover, neither NTG nor application of preconditioning stimuli was effective in increasing cardiac cGMP level in the tolerant state [10, 34, 35]. It was concluded that NTG might elicit cardioprotection without involvement of cGMP [29]. Contrary to these findings, clinical studies have suggested that long-term nitrate treatment does not improve or may even worsen cardiovascular mortality because nitrate tolerance has been shown to increase superoxide and peroxynitrate production leading to vascular dysfunction [12, 13, 23, 25, 36, 37]. These data are in accordance with our result-related hemodynamic events and more high incidence of VA and mortality in NTG-tolerant rats vs. nontolerant animals and correlate with findings of previous investigators that have reported increased expression of endothelin-1 in endothelial and smooth muscle cells, which in turn stimulates vascular superoxide production, resulting not only in increased endothelial-1 mediated vasoconstriction but also in increased formation of peroxynitrate [10]. Previous studies have suggested that NTG activates capsaicin-sensitive sensory nerves to release CGRP, which mediates the depressor effect and vasodilation produced by NTG via the cGMP pathway [14, 38, 39]. It has been demonstrated that the vasodilator responses and depressor effect of NTG are attenuated or abolished by CGRP antagonist CGRP_8-37_, or capsaicin, which selectively depletes CGRP in sensory nerves [9, 40]. Methylene blue, an inhibitor of soluble guanylate cyclase, abolishes the increased release of CGRP produced by NTG [40]. These findings support the hypothesis that the cardiovascular effect of nitroglycerin is, at least partially, mediated by endogenous CGRP via the cGMP pathway [14, 38]. The development of tolerance to NTG is associated with the desensitization of mitochondrial ALDH-2 [40], leading to an attenuated rise in cGMP, and NTG-mediated CGRP release through the cGMP pathway [14, 40]. These findings are in agreement with our results that showed the significant reduction of NTG-induced hemodynamic changes and BRS in NTG-tolerant rats + CGRP antagonist with the highest incidence of VA and mortality indicating an important role of CGRP in the development of NTG-induced tolerance and NTG effectiveness in AMI.

## Conclusions

Our results suggested that NTG tolerance may worsen its anti-ischemic effect in AMI and cardiovascular mortality, possibly due to the development of vascular desensitization involving alteration in CGRP, PGE_2_ production, and homeostatic hemodynamic mechanisms. They provide support for a concept that a stable CGRP agonist may have a stable preventive and therapeutic potential. The obtained results gave ground to personalized treatment with nitrates in various populations; for example, it could be considered that attenuation of nitric oxide-mediated vasodilation among blacks as compared to non-blacks is different throughout racial differences in synthesis and inactivation of endogenous bioactive nitric oxide.

## Recommendations

The therapy with nitrates in patients with cardiovascular disease requires the development of a personalized algorithm of treatment regime.

For avoiding the risk of reduction of CGRP releases, the intermitted regime of nitrate therapy could be recommended.

In the cohorts of patients with initial diminished levels of prostaglandin E_2_, the continuous treatment with nitrates could be indicated along with agents improving endothelial function.

## Abbreviations

AC: adenylyl cyclase
AMI: acute myocardial infarction
BP: blood pressure
BRS: baroreflex sensitivity
cGMP: cyclic guanosine monophosphate
CGRP: calcitonin gene-related peptide
CGRP_8-37_: human calcitonin gene-related peptide antagonist
HP: heart period
NTG: nitroglycerin
PGE_2_: prostaglandin E_2_
p-VASp: phosphorylation of vasodilator-stimulated phosphoprotein
ROS: reactive oxygen species
sGC: soluble guanylate cyclase
VA: ventricular arrhythmias
VF: ventricular fibrillation
VPBs: ventricular premature beats
VT: ventricular tachycardia
